# Homologous series by Nikolai Vavilov in the phylogeny of Homoptera

**DOI:** 10.3897/CompCytogen.v14i4.60894

**Published:** 2020-12-17

**Authors:** Ilya A. Gavrilov-Zimin

**Affiliations:** 1 Zoological Institute, Russian Academy of Sciences, Universitetskaya nab. 1, St. Petersburg, 199034, Russia Zoological Institute, Russian Academy of Sciences St. Petersburg Russia

**Keywords:** Aphids, cicadas, homologous variability, parallel evolution, psyllids, scale insects, whiteflies

## Abstract

The paper briefly discusses the most impressive examples of the Nikolai Vavilov’s “Law of homologous series” in the evolution of one of the largest animal groups, homopterous insects, which comprise about 65,000 recent species in the world fauna. Different taxonomic and phylogenetic characters (morpho-anatomical, cytogenetic, reproductive and others) are considered at the taxonomic ranks of the order, suborder, superfamily and family.

## Introduction

The famous geneticist and evolutionist Nikolai I. Vavilov (1887–1943) manifested his “Law of homologous series in variation” one hundred years ago (Vavilov 1920; Kolchinsky 2017; Bulatova 2020a). The Law was described as a universal rule which is applicable to all plants, animals and microorganisms, although, N.I. Vavilov was primarily a botanist and illustrated his findings mainly by examples from plant morphology, physiology and genetics. In view of this fact, it is not surprising that the Law was subsequently cited predominantly in the works of botanists, geneticists and historians interested in biology (see, for example, Goncharov 2014; Kolchinsky 2017). Zoological illustrations of the Law, although rather rare, could be found, for example, in the paper on the evolution of the morphological characters of Echinodermata in paleontological material (Rozhnov 2006), in the discussion of fur color variability in farm bred American mink (Trapezov 2007), in the article on natural chromosomal variability of the common shrew (Bulatova 2020b) and some others scientific publications. It also worth to mention, that some of the evolutional trends in the Animalia described with such terms as “arthropodization”, “ornithization”, “mammalization” (see for review Markov 2020) could also be considered in the frames of the Law of homologous series.

The present paper will briefly describe Vavilov’s Law of homologous series in the evolution of one of the largest animal groups, homopterous insects. The order Homoptera comprises about 65,000 recent species in the world fauna. It is subdivided into five recent suborders: Aphidinea (about 6,000 species), Coccinea (8,000 species), Psyllinea (3,500 species), Aleyrodinea (1,500 species) and Cicadinea (47,000 species). More detailed information about general classification and nomenclature of these taxonomic groups can be found, for example, in the monographs of Danzig and Gavrilov-Zimin (2014: 36) or Gavrilov-Zimin (2018: 21). There are also numerous publications addressing the phylogeny of these organisms. One of the main problems uniting majority of those publications is poor understanding of the differences between synapomorphic characters, inherited from the common ancestor, and parallelisms or homologous characters in N. Vavilov’s sense. The last characters evolve independently in the related organisms because their related genomes demonstrate similar response to a similar environment pressure.

## Homologous characters at the order level

Some authors (for example, Emeljanov 1987: 22) identify several morphological structures of the wing apparatus as synapomorphies of Cicadinea and true bugs (order Heteroptera). If those characters were really inherited by both groups from the common ancestor, it would be a proof of the paraphyly in the order Homoptera, which has Heteroptera evolved inside the group. Although, both Cicadinea and Heteroptera are characterized by deep adaptations for the perfect flight, all other suborders of Homoptera as well as the members of the related order Thysanoptera (thrips) have significantly reduced wing structure and are able to fly rather badly or not at all (for example, females of all 8,000 species of scale insects and many species of aphids completely lost their wings). In a view of this fact, it is not unlikely that the progressive adaptations of Cicadinea and Heteroptera to the flight evolved independently during the parallel evolution as a result of the homologous changes in the related groups of genes. Unfortunately, it is impossible now to unequivocally prove or reject this assumption without detailed molecular studies of the appropriate genes. Moreover, even the structure of the wing coupling, which is one of the characters, the most studied phylogenetically, is considered by some authors (for example, D’Urso 2002) in the opposite sense compared to the Emeljanov’s (l.c.) conclusions. Several additional morphological characters, not related to the wing apparatus, were also hypothesized as synapomorphies of Cicadinea and Heteroptera by Kluge (2020: 582–585). Unfortunately, those characters were studied on few exemplary species only and accepted with the different exclusions and stipulations, so, they, in my mind, cannot also be used for the characterization of such huge taxonomic groups as a whole.

**Figure 1.**
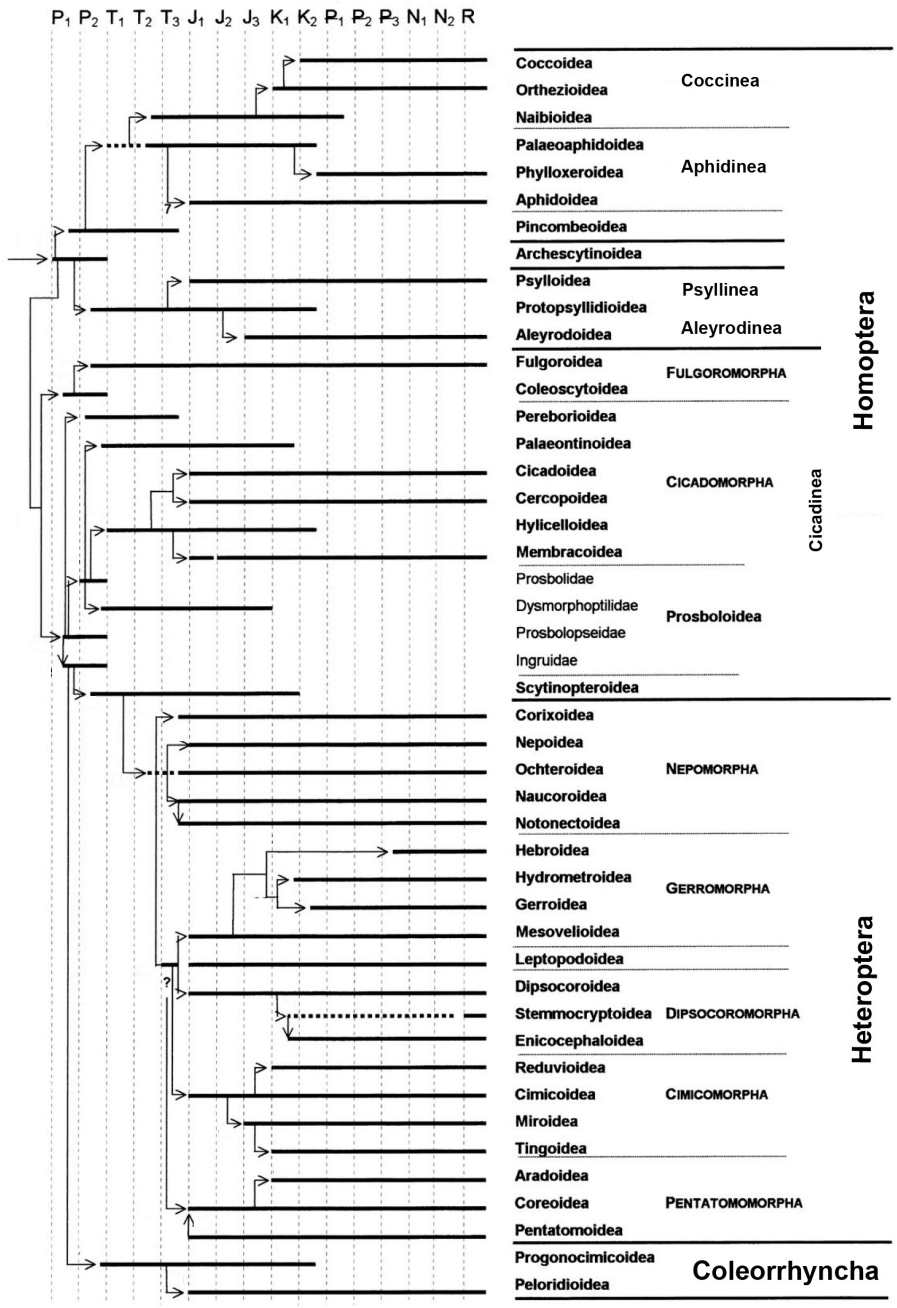
Reconstruction of the phylogeny of Homoptera and related insects, placed on geochronological scale (after Shcherbakov and Popov 2002: 146, with changes). Time periods **P_1_, P_2_** Early (Lower) and Late (Upper) Permian **T_1_, T_2_, T_3_** Early, Middle and Late Triassic **J_1_, J_2_, J_3_** Early, Middle and Late Jurassic **K_1_, K_2_** Early and Late Cretaceous ₽**_1_** Palaeocene ₽**_2_** Eocene ₽**_3_** Oligocene **N_1_** Miocene **N_2_** Pliocene **R** present time (Holocene).

On the other hand, the presence of the fields of wax glands, well-studied on large material in all archaic groups of Homoptera as well as in many younger groups of this order (see, for example, Šulc 1929; Emeljanov 2009; Danzig and Gavrilov-Zimin 2014, 2015; Gavrilov-Zimin 2018 and others), is undoubtedly an example of a good synapomorphic character, which is being not in direct connection with the adaptation to feeding on plant sup. The reliable proof of such phylogenetic assumption is the total absence of the homologous character (i.e., the fields of wax glands) in the numerous families of sap-sucking true bugs (Heteroptera) and thrips (Thysanoptera).

Another phylogenetically important question is the origin of the filter chamber in the digestive tract of Homoptera (see the review in Emeljanov 1987: 67), which is still remaining under discussion. The chamber was reported in most groups of Homoptera, but has not been found in any Heteroptera and Thysanoptera. That would be considered as an argument for the apomorphic origin of the chamber in Homoptera. On the other hand, the large variation of the fine structure of the filter chamber in different families may allude to the parallel homologous origin of this organ in accordance to Vavilov’s Law.

## Homologous characters in the suborders

Phylogenetic relationships among suborders of Homoptera are more or less understood now and the differences between synapomorphies and parallelisms are rather clear. It seems that all modern specialists (irrespective of their general theoretical views) agree with the close relationships in the following combination: Coccinea + Aphidinea, Psyllinea + Aleyrodinea and separately Cicadinea. The sequence of the evolutionary origin of these groups as well as the possible origin of one group within another still remain debatable among taxonomists (see, for example, discussion in Gavrilov-Zimin et al. 2015).

**Figure 2.**
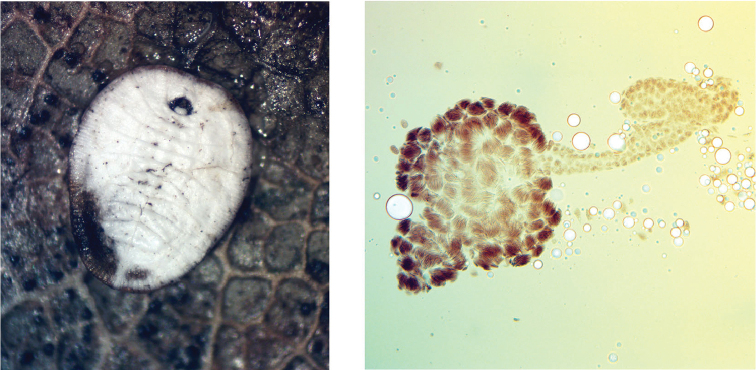
*Aleurochiton aceris* (Modeer, 1778) (Aleyrodinea), Russia (Moscow Prov.), male forth instar larva (pseudopuparium) and its testis with numerous bundles of sperm, produced in course of the larval meiosis.

Several examples of Vavilov’s Law at this taxonomic level could be mentioned: cytogenetic and ontogenetic parallelisms as a larval meiosis (known in scale insects, aphids and whiteflies, but unknown in psyllids) (Fig. 2), the appearance of the immovable and arostrate instars in the ontogenesis of whiteflies, aphids from the families Phylloxeridae and Pemphigidae, some achaeococcids and also in thrips, modal numbers of chromosomes are comparatively low in scale insects and aphids, etc. (see for more detail information: Gavrilov-Zimin et al. 2015; Gavrilov-Zimin 2018).

## Homologous characters in the superfamilies

**Figure 3.**
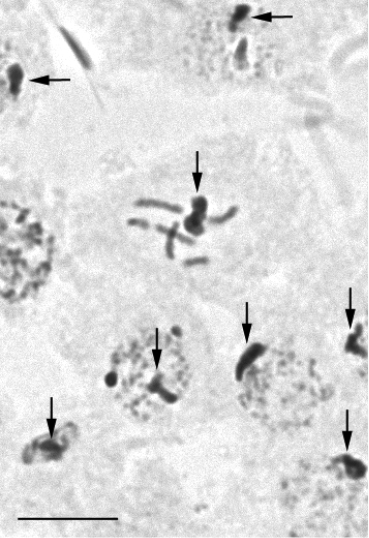
Heterochromatinization of paternal haploid set of chromosomes in the male embryonal cells of *Nipaeococcus delassusi* (Balachowsky, 1925) (Coccinea: Pseudococcidae), K 1276, Morocco (vicinity of Tangier); 2n = 12, heterochromatinized chromosomal sets are arrowed. Scale bar: 10 µm.

In accordance with Vavilov’s law, the number of homologous series increases significantly at lower taxonomic ranks. That is why, only examples primarily based on biology of scale insects, the group which is more familiar to the author, will be provided. The evolutionary advanced scale insect superfamily Coccoidea (so-called “neococcids”) is characterized by a peculiar heterochromatinization of paternal haploid set of chromosomes in males (Fig. 3). This heterochromatinization is usually considered as a synapomorphy of all neococcids (Danzig and Gavrilov-Zimin 2014), although this character is occasionally missing in some neococcid groups (for example, *Puto* Signoret, 1876 and *Stictococcus* Cockerell, 1903). This leaves a possibility of hypothetical parallel origin of heterochromatinization in different neococcid families. Moreover, very similar, but undoubtedly separately originated, heterochromatinization was found in some aphids of the family Lachnidae from the “advaced” aphid superfamily Aphidoidea (Blackman 1980) as well as in some Psocoptera (Hodson et al. 2017). Together, neococcids (Coccoidea) and both aphid superfamilies (Phylloxeroidea and Aphidoidea) demonstrate such rare parallelisms as physiological sex determination and formation of only two sperms (instead of four) from each primarily spermatocyte, whereas the basal scale insect superfamily Orthezioidea (archaeococcids) demonstrates XX-X0 sex-determination system with a normal producing of four sperms from each spermatocyte, usual for the most of insects (Gavrilov-Zimin et al. 2015).

## Homologous characters in the families

We (Danzig and Gavrilov-Zimin 2014; Gavrilov-Zimin 2018) accept 19 families of the scale insects in the world fauna. Almost all of these families show at least several unique morpho-anatomical and physiological peculiarities, which are unknown in any other animal group. Several characters in scale insects lead to external similarity in related, but clearly not sister groups of insects. Thus, a very unusual “dizygotic development”, which is similar in general appearance to the double fertilization of the flowering plants, is known for the members of the most archaic family of neococcids, Pseudococcidae, and also in the most evolutionary advanced family Diaspididae, but unknown in all other scale insect families. The presence of a paired symmetrical bacteriome is characteristic for some archaeococcids and for whiteflies (Aleyrodinea), whereas unpaired bacteriomes are characteristic for neococcids, aphids and psyllids (Buchner 1965). Most representatives of the family Pseudococcidae have so-called ostioles (one or two pairs of symmetrical openings on anterior and posterior segments of dorsum), which are probably homologous to siphunculi of aphids; the ventral abominal openings of some Pseudoccoidae (circuli) are probably homologous to marsupial opening in representatives of some genera of the archaeococcid family Margarodidae s.l. The cerarii, symmetrical groups of conical setae and wax glands along body margin of many Pseudococcidae, are clearly homologous with marginal tubercles of aphids, etc.

In many scale insect families, the whole “cycles of homologous variability” are to be observed in the accordance to the predictive modeling, based on Vavilov’s Law (1920). Thus, for example, the originally oviparous scale insects, evolved into marsupial or pseudomarsupial groups, already inside of the family Margarodidae s.l., which, in turn, gave the rise to the groups with a complete obligate ovoviviparity and then with an incomplete facultative ovoviviparity, when the oviposition of the slightly developed eggs occurs in the external wax ovisacs; in such a case, the secondary oviposition is almost restored in the course of evolution (Gavrilov-Zimin 2018). Similar evolutionary cycle is also repeated in different necococcid families. The aphids from the archaic superfamily Phylloxeroidea, are characterized by the normal oviparity, whereas the members of the more evolutionary advanced superfamily Aphidoidea, the reproductive mode evolves into the ovoviviparity and in the placental viviparity.

It is impossible to include all other homologous characters representing the evolution of wax glands, morphology of the anal apparatus, chaetotaxy, etc. in various scale insect families and genera; such comparison will require monographic treatment of numerous taxa. Many additional illustrations could be found, for example, in bi-volume book “Palaearctic mealybugs…” (Danzig and Garilov-Zimin 2014, 2015) and in the book on archaeococcids (Gavrilov-Zimin 2018).

To conclude, even this very brief review demonstrates difficulty in distinguishing Vavilov’s homologies from the cladistic synapomorphies in the order Homoptera. In some cases, the differences are clear and easily arguable, whereas in other examples, the hypothetical assumptions, basing on the current, often very limited knowledge of the subject, could only be provided. Taking this into consideration, Vavilov’s Law becomes a very uncomfortable factor in the practical work of the phylogeneticists and taxonomists, especially those who work in the field of the cladistic paradigm. Ideally, the researcher, when introducing a new character in phylogenetic analysis, should demonstrate not only the apomophic condition of this character in the putative sister taxa, but also he or she should prove that the character evolved only once in the hypothetical common ancestor, but not as a result of homologous variation in the related taxa. Such an approach would need long-time comprehensive study of each potential phylogenetic character in the numerous (ideally in all) species and genera of the analyzed higher taxon, which is, unfortunately, impossible in the frame of short-time projects, dominant now in modern day biology.
